# Incidental Pathogenic Fibrin-Associated Diffuse Large B-cell Lymphoma Found During Aorto-Biiliac Bypass

**DOI:** 10.7759/cureus.23681

**Published:** 2022-03-31

**Authors:** Peter M Habib, Thomas Serena, Caitlin M Flynn, Aaron Hartkop, Elizabeth Wey, David Lang, Eugene Laveroni

**Affiliations:** 1 Department of Surgery, Beaumont Health, Farmington Hills, USA; 2 Department of Internal Medicine, Beaumont Health, Farmington Hills, USA; 3 Department of Pathology, Beaumont Hospital, Royal Oak, USA

**Keywords:** aortoiliac disease, vascular surgery, aortobiliac bypass, ebv associated lymphoma, aortic lymphoma, fibrin-associated diffuse large b-cell lymphoma

## Abstract

Fibrin-associated diffuse large B-cell lymphoma (FA-DLBCL) is in and of itself a rare entity and is a subset of the Epstein-Barr virus (EBV)-associated lymphoma. Due to its indolent course, FA-DLBCL is generally an incidental finding on histopathological examinations. We present the first reported case of FA-DLBCL found within a native aortic thrombus during an aorto-biiliac bypass.

This is a 77-year-old male who was taken to the operative theater for open aorto-biiliac bypass secondary to aortooclusive disease resulting in intermittent claudication and gangrene of the right lower extremity digits. Intraoperatively, suspicious inflammatory changes were noted around the aorta. Pathological evaluation of the thrombus within the aorta noted cells of B-cell lineage with BCL2 and MYC positivity in addition to CD30 and EBV positivity. Postoperatively, the patient’s course was complicated by acute tubular necrosis, uremia, dialysis dependence, intubation, and cardiac arrhythmias including cardiac arrest. He was able to recover from these complications, however, he ultimately chose to self-enroll in hospice care.

An extensive literature review of over 128 mentions of FA-DLBCL noted a complete paucity of reported cases of FA-DLBCL within a native aorta. The patient’s clinical presentation and histopathology without mass-forming lesions lead to the diagnosis of FA-DLBCL. FA-DLBCL is an extremely rare EBV+ lymphoproliferative disorder associated with chronic inflammation (DLBCL-CI).

FA-DLBCL is a rare condition without defined uniform treatment. This article serves to highlight the first reported case of FA-DLBCL found within an abdominal aortic thrombus in a native aorta. Given the paucity of literature on this condition, postoperative treatment and long-term outcomes should be the focus of this condition.

## Introduction

Fibrin-associated diffuse large B-cell lymphoma (FA-DLBCL) is an extremely rare form of Epstein-Barr virus (EBV)-positive non-Hodgkin’s lymphoma. The authors present the case of a 77-year-old male claudicant who presented with atherosclerosis obliterans and new-onset digital ischemia. The patient underwent aorto-biiliac artery bypass with the intraoperative discovery of left common femoral artery thrombosis, requiring left femoral artery embolectomy with endarterectomy and patch angioplasty. Histological analysis of the aortic thrombus was consistent with an EBV+ lymphoproliferative disorder. Given the lack of mass-forming lesion, histological analysis of the aortic thrombus was consistent with FA-DLBCL. This case presents a rare case and unique presentation of a pathological non-Hodgkin’s lymphoma. This case was performed by community hospital vascular specialists and reported in accordance with the Surgical CAse REport SCARE Criteria and CARE Guidelines [1,2].

## Case presentation

A 77-year-old male presented to the vascular surgery clinic with complaints of worsening claudication, new-onset ischemia of toes, and decreased walking distance to 50 yards. The patient reported associated pain in the toes causing him to limp. He had been using lidocaine ointment on the toes for several weeks and noticed skin breakdown with an initial improvement in pain. The patient was well-known to the vascular surgery service with a longstanding history of claudication treated with dual-oral anti-platelet therapy and a history of previous right superficial femoral artery (SFA) occlusion treated with femoral endarterectomy and patch angioplasty three years prior to this visit. Past medical history was significant for obstructive sleep apnea with continuous positive airway pressure (CPAP) non-compliance, rheumatoid arthritis, pulmonary fibrosis, ischemic cardiomyopathy, type II diabetes, hypertension, coronary artery disease, and peripheral vascular disease. The patient was on chronic methotrexate and steroids for at least seven years prior to this visit. Past surgical history was significant for drug-eluting cardiac stenting and automatic implantable cardioverter-defibrillator placement 19 and 13 years prior to visit, respectively. The patient denied smoking history but did have significant exposure to secondary-hand smoke. Family history was non-contributory.

**Figure 1:**
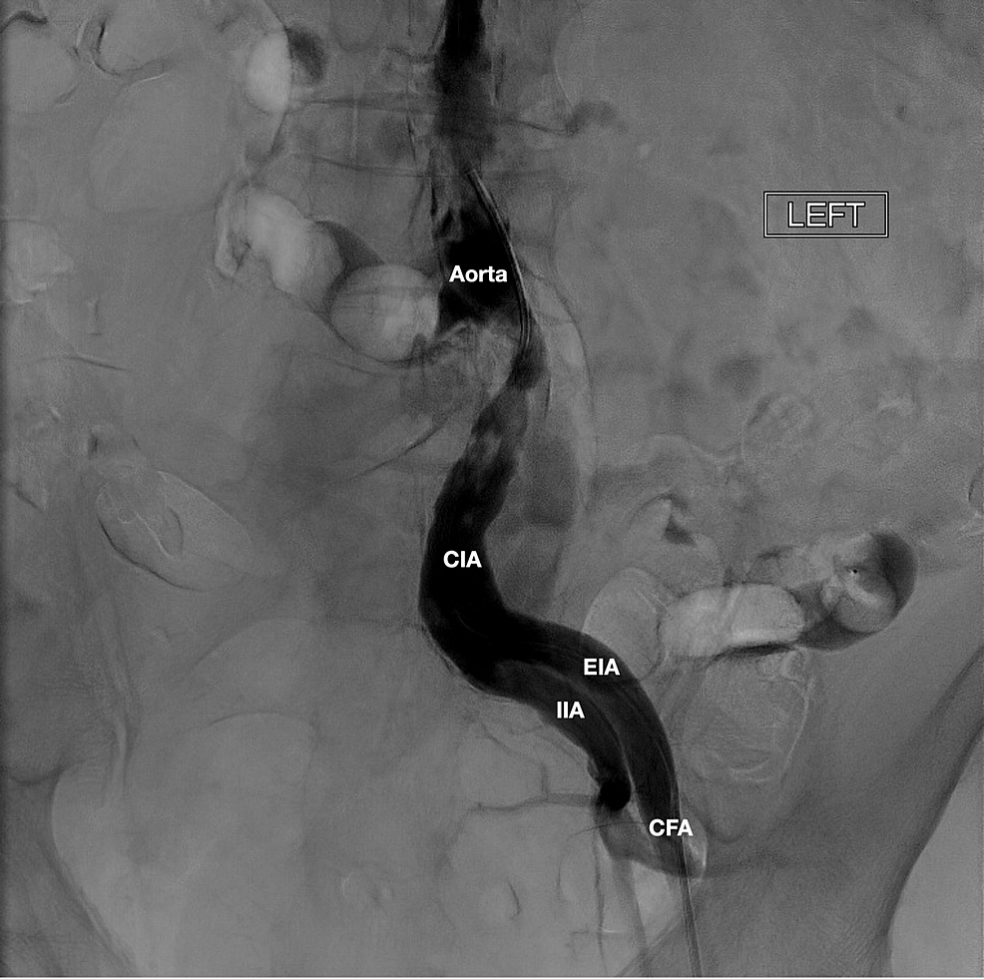
Invasive angiography of aorta with runoff demonstrating atherosclerotic changes in aorta, significant disease within the left CIA, and occlusion of the right CIA. CIA: common iliac artery; EIA: external iliac artery; IIA: internal iliac artery; CFA: common femoral artery

**Figure 2:**
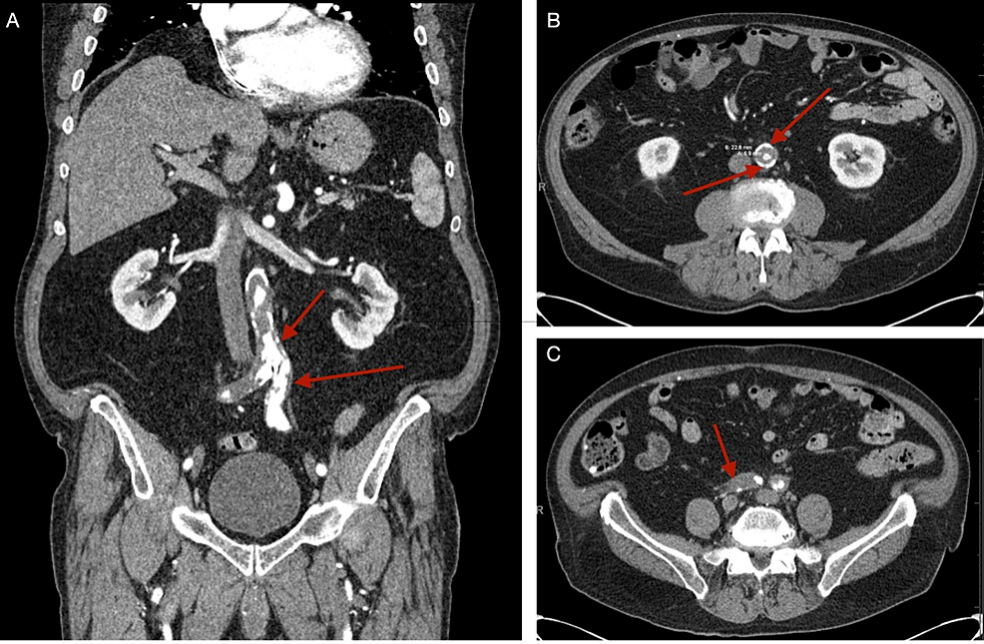
Computed tomography angiogram of the abdominal aorta with bilateral lower extremity runoff: A) Coronal view demonstrating extensive calcific and atherosclerotic disease of aorta extending into right common iliac artery. B) Axial view at level of kidneys demonstrating circumferential atheromatous plaquing noted within abdominal aorta resulting in luminal narrowing up to 70%. C) Axial view below aortic bifurcation demonstrating occlusion of the right common iliac artery.

Clinical examination revealed dry gangrene to all digits of the right lower extremity without evidence of lymphangitis, crepitus, or erythema. Popliteal, dorsalis pedis, and posterior tibial pulses were non-palpable bilaterally. The patient endorsed significant foot pain and altered gait. He had no leukocytosis. Hemoglobin was 10.0 g/dL. Creatinine was 1.2 mg/dL which had been his baseline for the past year. The patient was taken for conventional angiography that demonstrated atherosclerotic changes in the aorta, significant disease within the left common iliac artery (CIA), and occlusion of the right CIA (Figure 1). The results of invasive angiography prompted computed tomography angiogram of the abdominal aorta with bilateral lower extremity runoff revealing at least 70% luminal narrowing of the aorta from superficial mesenteric artery (SMA) to the aortic bifurcation, secondary to circumferential plaquing and thrombus. Imaging also demonstrated right CIA occlusion with reconstitution, atherosclerotic disease of the right SFA, occlusion of right anterior tibial (AT) artery, short segment severe stenosis of left proximal SFA, and distal SFA stenosis with occlusion of the left AT (Figure 2).

**Figure 3:**
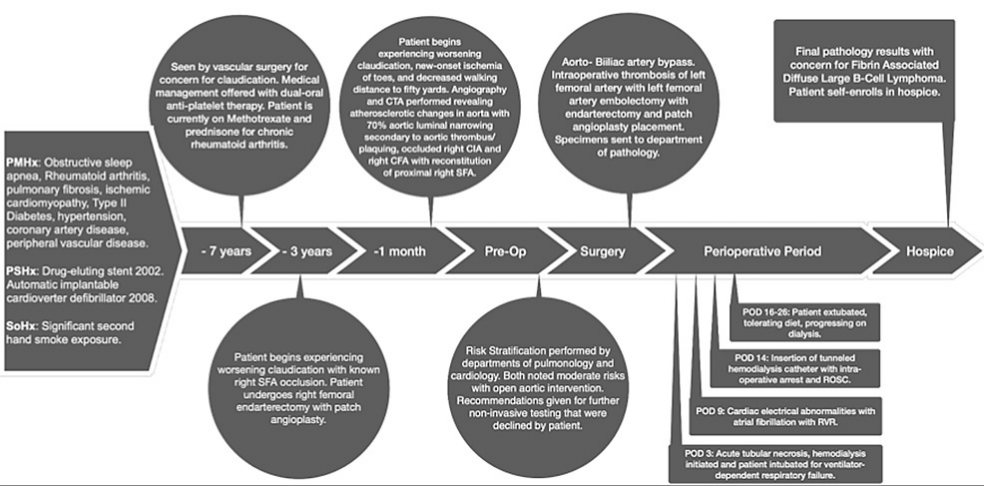
A chronology of notable events throughout patient history. PMHx : past medical history; PSHx: past surgical history; SoHx: social history; CTA: computed tomography angiography; SFA: superficial femoral artery; CIA: common iliac artery; CFA: common femoral artery; Pre-Op: preoperative period; POD: postoperative day; RVR: rapid ventricular response; ROSC: return of spontaneous circulation

With the progression of the patient’s disease process despite compliance with the best medical therapy, a discussion was held with the patient regarding his pathology and management options including but not limited to extra-anatomical and open aortic surgical intervention. Risk stratification was performed by departments of pulmonology and cardiology, both of which suggested moderate risks with open aortic intervention. Recommendations were given for further non-invasive testing that were declined by the patient. After weighing his options and multiple discussions regarding his elevated cardiac and pulmonary risk of open aortic repair, the patient opted for an open abdominal aortic bypass. A complete timeline of the patient’s preoperative and postoperative course can be found in Figure 3.

Intervention

Surgical Management

The patient was taken for exploratory laparotomy which was performed from xiphoid process to pubic symphysis. The bowel was retracted superiolaterally to the right of the patient exposing the root of the mesentery. The retroperitoneum was entered over the course of the aorta. Upon visualization of the aorta, significant inflammatory changes there were noted in periaortic tissue without signs of active infection. A decision was made to proceed with the bypass. The left renal vein, bilateral renal arteries, and suprarenal aorta were identified and creatine kinase (CK) circumferentially controlled. Distal control was obtained at bilateral internal and external iliac arteries. The patient was heparinized, and bilateral renal arteries, the suprarenal aorta, and bilateral iliac arteries were clamped. The infrarenal aorta was transected and extensive mural thrombus throughout the entirety of the infrarenal aorta as well as bilateral renal artery os was identified and excised en bloc and sent to pathology. A 20 × 10 mm Gore-tex graft was brought into the field and an aorto-biiliac bypass was performed. After all clamps had been removed, the patient had excellent pulses in bilateral femoral, bilateral external iliac, and bilateral renal arteries. The patient’s abdomen was then closed. When applying the final dressing, the quality of the pulse in the left femoral artery was poor and subsequently was lost. The patient was re-prepped and draped and underwent a left femoral endarterectomy with Fogarty thrombectomy of large iliac thrombus with patch angioplasty of the left common femoral artery. Following completion, the patient had palpable femoral and popliteal pulses bilaterally. The patient tolerated the procedure well and was transferred into recovery in stable condition.

Histological analysis

Pathological examination was performed on intraoperative thrombectomy and embolectomy samples. On gross inspection, the aortic thrombus material consisted of grossly unremarkable thrombus material with a scant vessel wall, without mass-forming lesions present. Histologically, lymphoma cells were arranged in thin delicate ribbons, lining the luminal surface of copious fibrinous debris and clot. The lymphoma cells were large with moderate cellular pleomorphism and displayed irregular nuclei with coarse chromatin, occasional prominent nucleoli, and moderate amounts of amphophilic cytoplasm. Multiple scattered apoptotic bodies and occasional mitoses were identified. Background reactive chronic inflammation was not seen within the fibrinous debris; however, scant chronic inflammation was present within sections of the vessel wall with only rare atypical lymphoid cells present.

**Figure 4:**
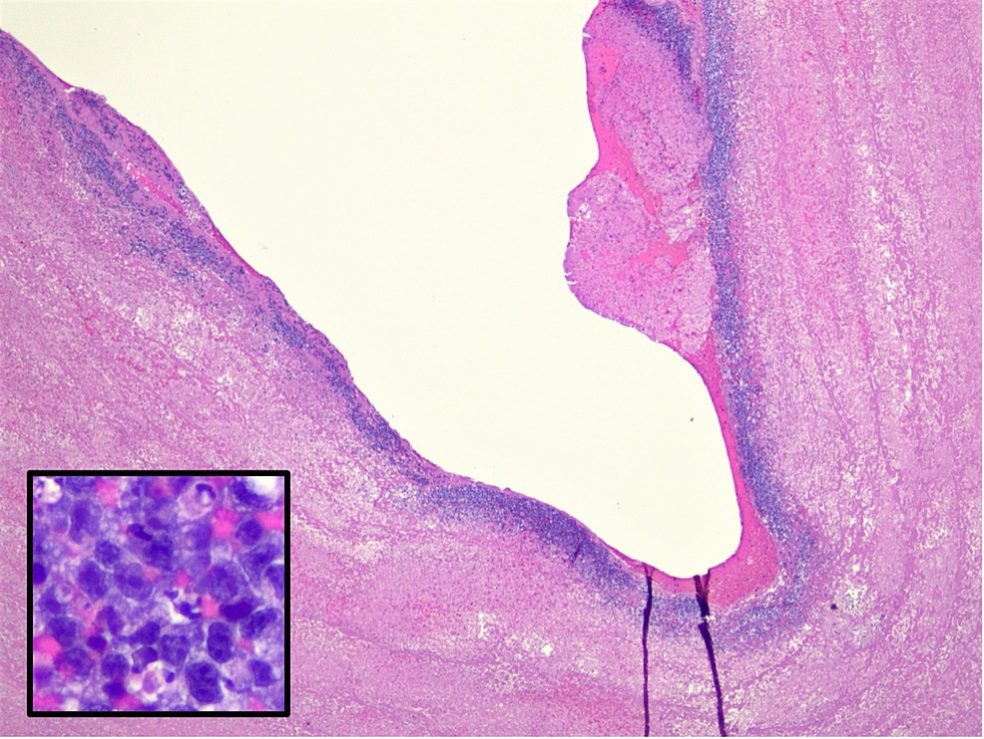
Histological examination of hematoxylin and eosin (H&E) (20×) and inset (400×): Histologic sections of the aortic thrombus consist of abundant fibrinous debris lined by thin ribbons of highly cellular tumor cells on the luminal surface (20×, H&E). On higher magnification, the viable tumor cells are densely packed, large pleomorphic lymphoid cells with irregular nuclei, prominent nucleoli, and amphophilic cytoplasm (400×, inset).

**Figure 5:**
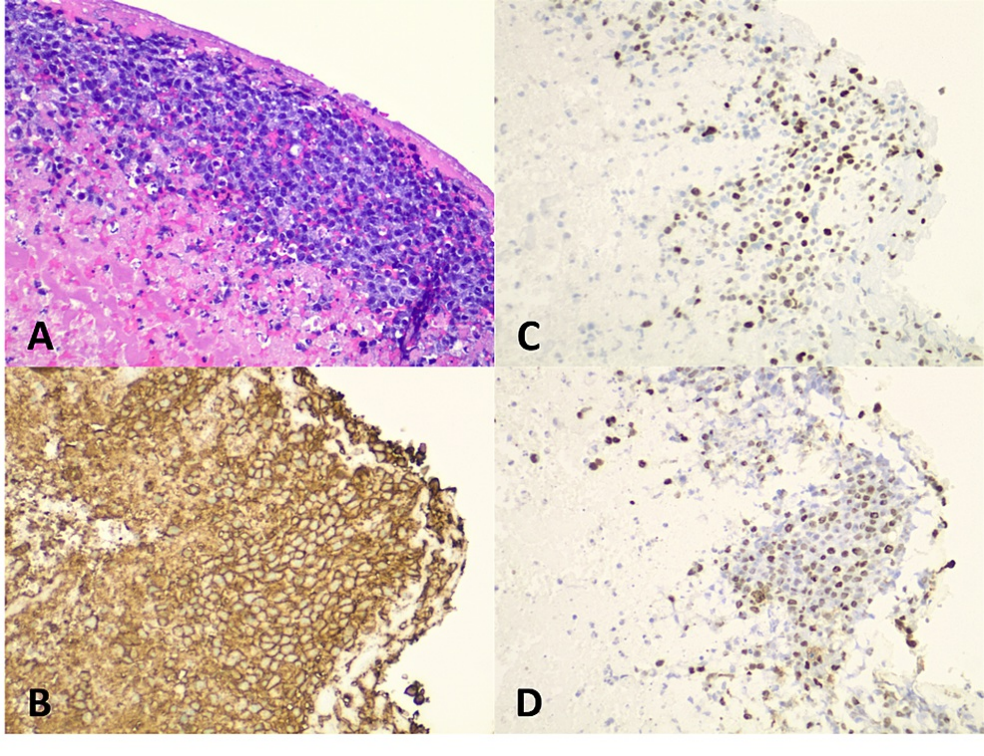
Histological immunostain composite image with H&E at 200×: A) H&E immunohistochemical staining reveals the large lymphoid cells. B) Positive staining for CD20 membrane-embedded surface molecule. C) Positive staining for MYC proto-oncogene. D) Positive staining for EBV by in-situ hybridization for EBER. H&E: hematoxylin and eosin; EBV: Epstein–Barr virus; EBER: Epstein-Barr encoding region

Immunohistochemical staining of the tumor cells revealed B-cell lineage (CD20 and PAX5 positive) of non-germinal center origin (CD10 negative, BCL6 positive, and MUM1 positive). T-cell-associated markers, CD3 and CD5, were negative. BCL2 and MYC (50% of nuclei) were also positive as were CD30 and EBV (Epstein-Barr virus) by in-situ hybridization for Epstein-Barr Encoding Region (EBER). The Ki67 revealed a proliferative index of approximately 95%. Fluorescence in-situ hybridization (FISH) studies performed on the paraffin block were positive for MYC gene rearrangement in 27.5% of nuclei. Further testing revealed no IGH/MYC or IGL/MYC gene rearrangements. There were no BCL2 or BCL6 gene rearrangements (no evidence of “double-hit” lymphoma); however, there was apparent loss of BCL2 (35% nuclei) and BCL6 (30% nuclei) genes (Figures 4-5).

Postoperative course

The patient’s immediate postoperative course was uneventful until postoperative day three when the patient developed acute tubular necrosis and hemodialysis was initiated. In addition, the patient developed uremic encephalopathy and was intubated for airway protection. The patient remained in the intensive care unit secondary to difficulties with ventilator weaning and he eventually manifest cardiac electrical abnormalities with atrial fibrillation and rapid ventricular response. Final intraoperative pathology resulted and findings consistent with FA-DLBCL in the context of a lack of mass-forming lesions. Extensive discussions were had regarding lifelong dialysis and his new diagnosis of non-Hodgkin’s lymphoma at which point the patient opted to self-enroll in hospice and subsequently passed away.

## Discussion

**Figure 6:**
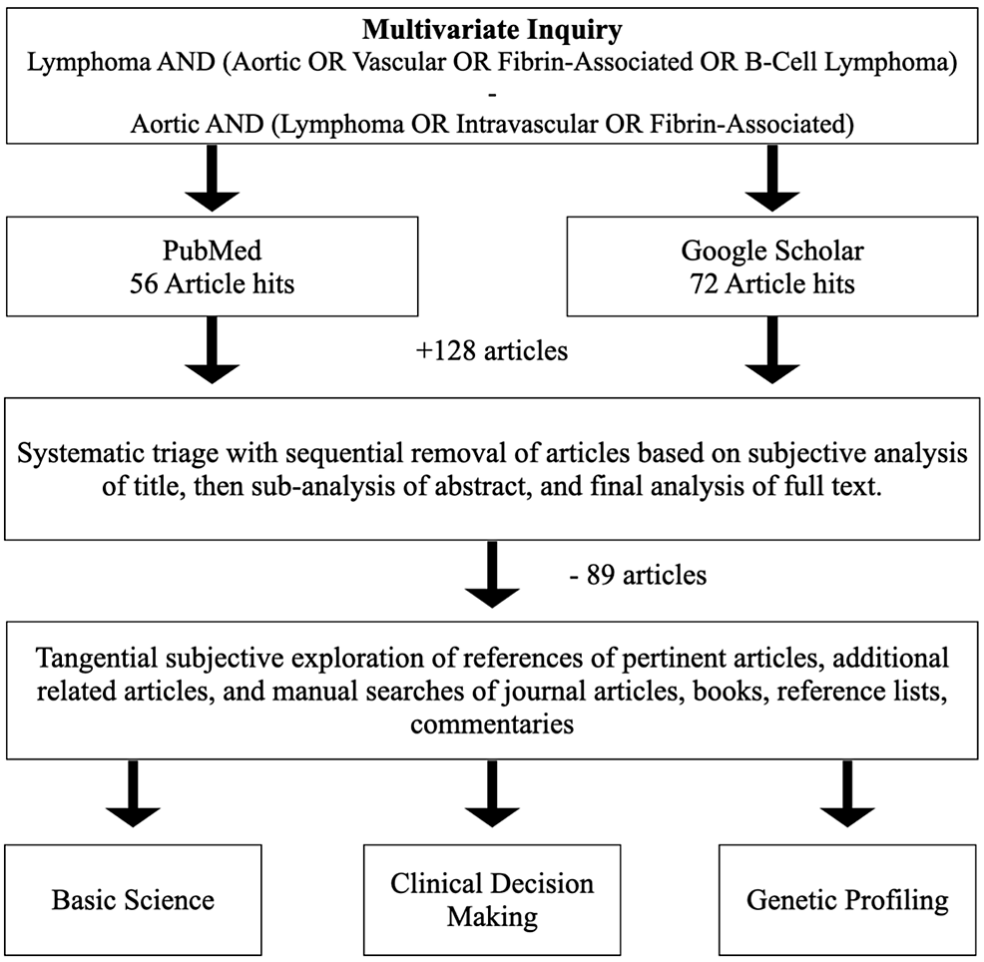
Flowchart depicting methods for multivariate inquiry literature review.

A comprehensive electronic database search was performed in PubMed, Google Scholar, and referenced manuscripts to identify all the articles that reported on fibrin-associated aortic lymphomas. The search was not limited to the time or language of the published study. The primary endpoints of this review included basic science research, genetics, surgical management, and clinical data outlining the progression of the disease. Multivariate inquiry (as described in Figure 6) was performed with base searches for “lymphoma” crossed with key phrases of “aortic, vascular, fibrin-associated” and also “aortic” crossed with key phrases of “lymphoma, intravascular, and fibrin-associated”. This inquiry resulted in 128 published articles describing fibrin- FA-DLBCL or intravascular B-cell lymphoma (IVBCL), none of which mentioned FA-DLBCL found in the abdominal aortic thrombus. The sole case most similar was found to involve a patient with a history of B-cell lymphocytosis and prior aortic root graft placement who was found to have thrombus in said graft. Thus, this incidental finding of FA-DLBCL in an abdominal aortic thrombus of a native abdominal aorta appears to be unique and previously unreported [3].

Intravascular large BCL has been described as a rare entity with exclusive growth of large cells within the lumen of all sized blood vessels [4]. As with all BCLs, patients may present with classic B-symptoms such as night sweats, fever, weight loss, and fatigue. However, IVLBCL patient presentation is specific to the subtype and manifestation of the disease process and location. Subtypes of IVLBCL include 1) intravascular large BCL classical, 2) intravascular large BCL, cutaneous variant, and 3) intravascular large BCL, hemophagocytic syndrome-associated variant. One report presented an immunocompetent woman with cerebral aneurysm rupture and incidental FA-DLBCL identified within the thrombotic material. In their literature review, they noted that FA-DLBCL arises as small foci of neoplastic cells within the fibrinous materials in the context of hematomas, pseudocyst, cardiac myxomas, or in relation to prosthetic devices [5].

To reiterate from earlier in the text, gross inspection of the presented patient’s thrombus revealed no mass-forming lesion nor did his systemic clinical and radiographic workup. Histopathologic examination of the aortic thrombus specimen demonstrated thin delicate ribbons of lymphoma cells lining the luminal surface of the fibrous debris and clot with B-cell lineage (CD20 and PAX5 positivity) with a non-germinal center B-cell phenotype, strong positivity for EBV-encoded RNA, and a Ki-67 proliferative rate of ~95%. All of the aforementioned are consistent with FA-DLBCL given the presence of fibrin and lack of tumor formation.

EBV is a very common global infection with an estimated 80-95% of adults being seropositive [6]. FA-DLBCL is an extremely rare form of EBC-positive lymphoma. It has been classified by the WHO as a subset entity of diffuse large BCL associated with chronic inflammation (DLBCL-CI). This specific non-Hodgkin’s lymphoma is unique in that it is not mass-forming and has a more favorable prognosis, in stark contrast to notoriously aggressive DLBCL-CI. Furthermore, it is almost exclusively found in patients’ post-surgical histological evaluation rather than during preoperative workup. Treatment, while non-uniform, typically consists of exclusive surgery. Some reported cases have documented adjuvant chemotherapy, radiation, or immunologic therapy. Local recurrence or persistent disease has only been shown in primary sites of atrial myxoma and prior grafts [5]. Further research is required to determine best practices in the management of this condition.

Subcategories of EBV+ lymphoproliferative disorders are diffuse large BCL associated with chronic inflammation (DLBCL-CI) and diffuse large BCL, not otherwise specified (DLBCL-NOS). Both have an elderly age distribution with a predominance of the male sex. Histologically, DLBCL-CI is often plasmablastic or immunoblastic, whereas EBV+ DLBCL-NOS is typically polymorphic [5]. Both are CD20 positive and CD10 negative. EBV+ DLBCL-NOS is a diagnosis of exclusion. DLBCL-CI is found in patients with chronic, longstanding inflammatory states such as chronic osteomyelitis, metallic implant, and chronic skin ulcers [6]. This patient’s rheumatoid arthritis and the induced iatrogenic immunosuppression may be linked to his diagnosis. Recently, FA-DLBCL has been proposed to become a separate entity from DLBCL-CI given the lack of mass formation, indolent clinical course, and better overall prognosis in FA-DLBCL. Histologically, FA-DLBCL cells are of non-germinal center origin and found to be arranged in a single line or in small clusters within a fibrinous material. FA-DLBCL tumor cells have a high proliferative index (Ki-67>90%) [7]. Upon literature review, we have found this to be the first reported case of FA-DLBCL found within an abdominal aortic thrombus of a native aorta, consistent with the most recent sub-classification by WHO, and further promotes the notion that FA-DLBCL should be recategorized as its own separate entity.

There are multiple limitations in the analysis of this patient’s course and condition including a lack of positron emission tomography and bone marrow biopsy. Without complete workup, the final pathological diagnosis could be subject to debate given unknown staging or synchronous lesions. The overall prognosis and long-term survival of our patient are also limited by the patient’s decision to self-enroll in hospice. In addition, given the rarity of the patient’s condition, there is limited power to any information on the long-term prognosis and characteristics of this pathology. A strength of this report is the extensive evaluation of all available literature. Furthermore, the greatest strength of this study is that this is the first known presentation of an intra-aortic FA-DLBCL.

## Conclusions

FA-DLBCL is a rare condition. Given the rarity of this condition, treatment varies with most patients undergoing only surgical excision with a smaller portion receiving adjuvant therapies. This article serves to highlight the first reported case of FA-DLBCL found within an abdominal aortic thrombus in a native aorta. Given the rarity of this condition, further research should center on postoperative treatment and long-term outcomes.
